# Supplementary material for: Migration of gastric cancer is suppressed by recombinant Newcastle disease virus (rL-RVG) via regulating α7-nicotinic acetylcholine receptors/ERK- EMT
Source: BMC Cancer. 2019 Oct 22;19:976. doi: 10.1186/s12885-019-6225-9 (PMC6805660; doi:10.1186/s12885-019-6225-9)
Supplement: Supplementary file 1 — Additional file 1: Result of STR matching analysis by your data. [file 12885_2019_6225_MOESM1_ESM.pdf]

Result of STR matching analysis by your data.

- DSMZ Profile Database -

A graphical presentation is shown at the bottom of this page.

EV	Cell No.	Cell name	D5S818	D13S317	D7S820	D16S539	VWA	TH01	AM	TPOX	CSF1PO	Figures
		Query (Your Cell)	*11,12*	*13.3,13.3*	*12,12*	*9,10*	*16,18*	*7,7*	*X,X*	*12,12*	*10,10*	
0.89 (32/36)	CCL-21	AV3	11,12	13.3,13.3	12,12	9,10	16,18	7,7	X,X	8,12	9,10	-
0.83 (30/36)	227	BT-B	11,12	13.3,13.3	8,12	9,10	16,18	7,7	X,X	8,12	9,10	-
0.83 (30/36)	228	SBC-2	11,12	12,13.3	12,12	9,10	16,18	7,7	X,X	8,12	9,10	-
0.83 (30/36)	229	SBC-7	11,12	12,13.3	12,12	9,10	16,18	7,7	X,X	8,12	9,10	-
0.83 (30/36)	CCL-2.2	HeLa S3	11,12	13.3,13.3	8,12	9,10	16,18	7,7	X,X	8,12	9,10	-
0.83 (30/36)	CCL-25	WISH	11,12	13.3,13.3	8,12	9,10	16,18	7,7	X,X	8,12	9,10	-
0.83 (30/36)	CRL-13003	GH354	11,12	13.3,13.3	8,12	9,10	16,18	7,7	X,X	8,12	9,10	-
0.83 (30/36)	IFO50004	WISH	11,12	13.3,13.3	8,12	9,10	16,18	7,7	X,X	8,12	9,10	-
0.83 (30/36)	JCRB0213	HeLa AG	11,12	13.3,13.3	8,8	9,10	16,18	7,7	X,X	12,12	9,10	-
0.83 (30/36)	JCRB0214	HeLa TG	11,12	13.3,13.3	8,8	9,10	16,18	7,7	X,X	12,12	9,10	-
0.83 (30/36)	JCRB0215	HeLa TG CAP	11,12	13.3,13.3	8,8	9,10	16,18	7,7	X,X	12,12	9,10	-
0.83 (30/36)	JCRB0649.1	HeLa.P3	11,12	12,13.3	8,12	9,10	16,18	7,7	X,X	12,12	9,10	-
0.80 (28/35)	RCB2105	D98-AH2	11,12		12,12	9,10	16,18	7,7	X,X	8,12	9,10,11	-
0.78 (28/36)	57	HELA	11,12	12,13.3	8,12	9,10	16,18	7,7	X,X	8,12	9,10	-
0.78 (28/36)	57	HELA	11,12	12,13.3	8,12	9,10	16,18	7,7	X,X	8,12	9,10	-
0.78 (28/36)	116	GIRARDI HEART C2	11,12	12,13.3	8,12	9,10	16,18	7,7	X,X	8,12	9,10	-
0.78 (28/36)	121	GIRARDI HEART C7	11,12	12,13.3	8,12	9,10	16,18	7,7	X,X	8,12	9,10	-
0.78 (28/36)	136	KB	11,12	12,13.3	8,12	9,10	16,18	7,7	X,X	8,12	9,10	-
0.78 (28/36)	149	KB-V1	11,12	12,13.3	8,12	9,10	16,18	7,7	X,X	8,12	9,10	-
0.78 (28/36)	158	KB-3-1	11,12	12,13.3	8,12	9,10	16,18	7,7	X,X	8,12	9,10	-
0.78 (28/36)	161	HELA-S3	11,12	12,13.3	8,12	9,10	16,18	7,7	X,X	8,12	9,10	-
0.78 (28/36)	CCL-13	HeLa [Chang Liver]	12,12	12,13.3	8,12	9,10	16,18	7,7	X,X	8,12	10,10	-
0.78 (28/36)	CCL-17	KB	11,12	12,13.3	8,12	9,10	16,18	7,7	X,X	8,12	9,10	-
0.78 (28/36)	CCL-2	HeLa	11,12	12,13.3	8,12	9,10	16,18	7,7	X,X	8,12	9,10	-
0.78 (28/36)	CCL-2.1	HeLa 229	11,12	12,13.3	8,12	9,10	16,18	7,7	X,X	8,12	9,10	-
0.78 (28/36)	CCL-20.2	clone 1-5c-4 [Wong-Kilbourne derivative (D) of Chang conjunctiva]	11,12	12,13.3	8,12	9,10	16,18	7,7	X,X	8,12	9,10	-
0.78 (28/36)	CCL-23	HEp-2	11,12	12,13.3	8,12	9,10	16,18	7,7	X,X	8,12	9,10	-
0.78 (28/36)	CCL-5	L-132	11,12	12,13.3	8,12	9,10	16,18	7,7	X,X	8,12	9,10	-
0.78 (28/36)	CCL-6	intestine 407	11,12	12,13.3	8,12	9,10	16,18	7,7	X,X	8,12	9,10	-
0.78 (28/36)	CCL-62	FL	11,12	12,13.3	8,12	9,10	16,18	7,7	X,X	8,12	9,10	-

0.78 (28/36)	CL-48	WRL 68	11,12	12,13.3	8,12	9,10	16,18	7,7	X,X	8,12	9,10	-
0.78 (28/36)	CRL-12510	HeLa NRI	11,12	12,13.3	8,12	9,10	16,18	7,7	X,X	8,12	9,10	-
0.78 (28/36)	CRL-13002	GH329	11,12	12,13.3	8,12	9,10	16,18	7,7	X,X	8,12	9,10	-
0.78 (28/36)	CRL-13011	HeLaNR1	11,12	12,13.3	8,12	9,10	16,18	7,7	X,X	8,12	9,10	-
0.78 (28/36)	CRL-1958	H1HeLa	11,12	12,13.3	8,12	9,10	16,18	7,7	X,X	8,12	9,10	-
0.78 (28/36)	CRL-2972	HeLaRC32 [HeRC32]	11,12	12,13.3	8,12	9,10	16,18	7,7	X,X	8,12	9,10	-
0.78 (28/36)	CRM-CCL-2	HeLa	11,12	12,13.3	8,12	9,10	16,18	7,7	X,X	8,12	9,10	-
0.78 (28/36)	IFO50005	J-111	11,12	12,13.3	8,12	9,10	16,18	7,7	X,X	8,12	9,10	-
0.78 (28/36)	IFO50016	Chang Liver	11,12	12,13.3	8,12	9,10	16,18	7,7	X,X	8,12	9,10	-
0.78 (28/36)	JCRB0073	J-111	11,12	12,13.3	8,12	9,10	16,18	7,7	X,X	8,12	9,10	-
0.78 (28/36)	JCRB0649	HeLa.P3	11,12	12,13.3	8,12	9,10	16,18	7,7	X,X	8,12	9,10	-
0.78 (28/36)	JCRB1309	RPMI4788	11,12	13.3,13.3	8,12	10,10	16,18	7,7	X,X	8,12	9,10	-
0.78 (28/36)	JCRB1318	HeLa9903	11,12	12,13.3	8,12	9,10	16,18	7,7	X,X	8,12	9,10	-
0.78 (28/36)	JCRB9004	HeLa	11,12	12,13.3	8,12	9,10	16,18	7,7	X,X	8,12	9,10	-
0.78 (28/36)	JCRB9027	KB	11,12	12,13.3	8,12	9,10	16,18	7,7	X,X	8,12	9,10	-
0.78 (28/36)	JCRB9086	HeLa229	11,12	12,13.3	8,12	9,10	16,18	7,7	X,X	8,12	9,10	-
0.76 (26/34)	RCB0191	HeLa.S3	11,12		8,12	9,10	16,18	7,7	X,X	8,12	9,10	-
0.76 (26/34)	RCB0205	BU25 TK-	11,12		8,12	9,10	16,18	7,7	X,X	8,12	9,10	-
0.76 (26/34)	RCB1525	HeLa.S3	11,12		8,12	9,10	16,18	7,7	X,X	8,12	9,10	-
0.76 (26/34)	RCB1891	HeLa TG	11,12		8,8	9,10	16,18	7,7	X,X	12,12	9,10	-
0.76 (28/37)	JCRB9010	HeLa S3	11,12	13.3,13.3	8,8	9,10	16,17,18	7,7	X,X	8,12	9,10	-
0.76 (28/37)	JCRB9066	Chang Liver	11,12	12,13.3	8,12	9,10	16,17,18	7,7	X,X	8,12	9,10	-
0.72 (26/36)	RCB0007	HeLa	11,12	12,12	8,12	9,10	16,18	7,7	X,X	8,12	9,10	-
0.72 (26/36)	RCB0402	"HeLa¡¤P3"	11,12	12,12	8,12	9,10	16,18	7,7	X,X	8,12	9,10	-
0.72 (26/36)	RCB1889	HEp-2	11,12	12,12	8,12	9,10	16,18	7,7	X,X	8,12	9,10	-
0.72 (26/36)	RCB2356	HeLa-CD4-LTR-??-gal	11,12	12,12	8,12	9,10	16,18	7,7	X,X	8,12	9,10	-
0.72 (26/36)	RCB2358	HLtat	11,12	12,12	8,12	9,10	16,18	7,7	X,X	8,12	9,10	-
0.70 (26/37)	CRL-2182	EPLC-32M1	11,12	13.3,14.3	8,12	9,10	17,18	7,7	X,X	8,12,13	9,10	-
0.67 (24/36)	CHB0001	HSY	11,11	12,13.3	8,12	9,10	16,18	7,7	X,X	8,12	9,9	-
0.67 (24/36)	IFO50011	HeLa S3	11,11	12,13.3	8,12	9,10	17,18	7,7	X,X	8,12	9,10	-
0.67 (24/36)	JCRB0713	HeLa S3(sc)	11,11	12,13.3	8,12	9,10	17,18	7,7	X,X	8,12	9,10	-
0.67 (24/36)	RCB2355	HeLa CD4+Clone1022	11,12	12,12	8,12	9,9	16,18	7,7	X,X	8,12	9,10	-
0.65 (24/37)	CHB0002	HSG-AZA3	11,11	12,13.3	8,12	9,10	16,18,19	7,7	X,X	8,12	9,9	-
0.65 (24/37)	CHB0003	HSG-AZA1	11,11	12,13.3	8,12	9,10	16,18,19	7,7	X,X	8,12	9,9	-
0.65 (24/37)	CHB0004	HSG	11,11	12,13.3	8,12	9,10	16,18,19	7,7	X,X	8,12	9,9	-
	JCRB1070	HSGc-C5	11,11	12,13.3	8,12	9,10	16,18,19	7,7	X,X	8,12	9,9	-

0.65 (24/37)												
0.65 (24/37)	JCRB1070	HSGc-C5	**11**,11	12,**13.3**	8,**12**	**9**,**10**	**16**,**18**,19	**7**,**7**	**X**,**X**	8,**12**	9,9	-
0.65 (22/34)	RCB0503	HeLa.S3(Mer^(-))	**11**,**12**		8,8	**9**,**10**	15,**18**	**7**,**7**	**X**,**X**	8,**12**	9,**10**	-
0.65 (22/34)	RCB0504	MR1-3	**11**,**12**		8,8	**9**,**10**	15,**18**	**7**,**7**	**X**,**X**	8,**12**	9,**10**	-
0.65 (22/34)	RCB0507	MR10-1	**11**,**12**		8,8	**9**,**10**	15,**18**	**7**,**7**	**X**,**X**	8,**12**	9,**10**	-
0.63 (22/35)	RCB0505	MR6	**11**,**12**		8,8	**9**,**10**	15,**16**,**18**	**7**,9.3	**X**,**X**	8,**12**	9,**10**	-
0.61 (22/36)	367	BFTC-909	**11**,**12**	11,13	**12**,**12**	**9**,**10**	14,**18**	**7**,**7**	**X**,**X**	8,8	12,13	-
0.61 (22/36)	CRL-7164	Hs 203.Sp	**11**,**12**	8,9	**12**,**12**	11,11	**16**,**18**	**7**,**7**	**X**,**X**	8,11	**10**,12	-
0.61 (22/36)	CRL-7165	Hs 203.Th	**11**,**12**	8,9	**12**,**12**	11,11	**16**,**18**	**7**,**7**	**X**,**X**	8,11	**10**,12	-
0.61 (22/36)	RCB0271	HeLa S3 (SC)	**11**,11	12,12	8,**12**	**9**,**10**	17,**18**	**7**,**7**	**X**,**X**	8,**12**	9,**10**	-

---

Analyze again **after changing the EV** 0.60

EV	Cell No. Scored	
**0.95**~1.00	0	
**0.90**~0.95	0	
**0.85**~0.90	1	|
**0.80**~0.85	12	||||||||||||
**0.75**~0.80	39	|||||||||||||||||||||||||||||||||||||||
**0.70**~0.75	6	||||||
**0.65**~0.70	12	||||||||||||
**0.60**~0.65	5	|||||
0.55~0.60	14	||||||||||||||
0.50~0.55	59	
0.45~0.50	11	|||||||||||
0.40~0.45	141	
0.35~0.40	345	
0.30~0.35	713	
0.25~0.30	721	
0.20~0.25	649	
0.15~0.20	421	
0.10~0.15	106	
0.05~0.10	9	|||||||||
0.00~0.05	10	||||||||||

If the results obtained by the search engine are used in any publication, please cite the respective paper: Dirks WG, MacLeod RA, Nakamura Y, Kohara A, Reid Y, Milch H, Drexler HG, Mizusawa H.: Cell line cross-contamination initiative: an interactive reference database of STR profiles covering common cancer cell lines. Int J Cancer. 2010 Jan 1;126(1):303-4. (link: https://onlinelibrary.wiley.com/doi/full/10.1002/ijc.24999)
